# The Effects of Remote Cognitive Training Combined With a Mobile App Intervention on Psychosis: Double-Blind Randomized Controlled Trial

**DOI:** 10.2196/48634

**Published:** 2023-11-13

**Authors:** Melissa Fisher, Kevin Etter, Aimee Murray, Neelu Ghiasi, Kristin LaCross, Ian Ramsay, Ariel Currie, Karrie Fitzpatrick, Bruno Biagianti, Danielle Schlosser, Rachel Loewy, Sophia Vinogradov

**Affiliations:** 1 Department of Psychiatry & Behavioral Sciences University of Minnesota Minneapolis, MN United States; 2 Department of Psychiatry & Behavioral Sciences University of California, San Francisco San Francisco, CA United States; 3 Edgewood Center for Children and Families San Francisco, CA United States; 4 Department of Psychology University of Milan Bicocca Milano Italy

**Keywords:** schizophrenia, psychosis, cognitive training, motivation, mobile intervention, mobile phone

## Abstract

**Background:**

Impairments in cognition and motivation are core features of psychosis and strong predictors of social and occupational functioning. Accumulating evidence indicates that cognitive deficits in psychosis can be improved by computer-based cognitive training programs; however, barriers include access and adherence to cognitive training exercises. Limited evidence-based methods have been established to enhance motivated behavior. In this study, we tested the effects of web-based targeted cognitive and social cognitive training (TCT) delivered in conjunction with an innovative digital smartphone app called Personalized Real-Time Intervention for Motivational Enhancement (PRIME). The PRIME app provides users with a motivational coach to set personalized goals and secure social networking for peer support.

**Objective:**

This study investigated whether deficits in cognition and motivation in people with a psychosis spectrum disorder (N=100) can be successfully addressed with 30 hours of TCT+PRIME as compared with 30 hours of a computer games control condition (CG) plus PRIME (CG+PRIME). Here, we describe our study procedures, the feasibility and acceptability of the intervention, and the results on all primary outcomes.

**Methods:**

In this double-blind randomized controlled trial, English-speaking participants completed all cognitive training, PRIME activities, and assessments remotely. Participants completed a diagnostic interview and remote cognitive, clinical, and self-report measures at baseline, posttraining, and at a 6-month follow-up.

**Results:**

This study included participants from 27 states across the United States and 8 countries worldwide. The study population was 58% (58/100) female, with a mean age of 33.77 (SD 10.70) years. On average, participants completed more than half of the cognitive training regimen (mean 18.58, SD 12.47 hours of training), and logged into the PRIME app 4.71 (SD 1.58) times per week. The attrition rate of 22% (22/100) was lower than that reported in our previous studies on remote cognitive training. The total sample showed significant gains in global cognition (*P*=.03) and attention (*P*<.001). The TCT+PRIME participants showed significantly greater gains in emotion recognition (*P*<.001) and global cognition at the trend level (*P*=.09), although this was not statistically significant, relative to the CG+PRIME participants. The total sample also showed significant improvements on multiple indices of motivation (*P*=.02-0.05), in depression (*P*=.04), in positive symptoms (*P*=.04), and in negative symptoms at a trend level (*P*=.09), although this was not statistically significant. Satisfaction with the PRIME app was rated at 7.74 (SD 2.05) on a scale of 1 to 10, with higher values indicating more satisfaction.

**Conclusions:**

These results demonstrate the feasibility and acceptability of remote cognitive training combined with the PRIME app and that this intervention can improve cognition, motivation, and symptoms in individuals with psychosis. TCT+PRIME appeared more effective in improving emotion recognition and global cognition than CG+PRIME. Future analyses will test the relationship between hours of cognitive training completed; PRIME use; and changes in cognition, motivation, symptoms, and functioning.

**Trial Registration:**

ClinicalTrials.gov NCT02782442; https://clinicaltrials.gov/study/NCT02782442

## Introduction

### Background

Psychosis spectrum disorders are not only characterized by positive symptoms such as hallucinations and delusions but also by mood, motivational, and cognitive impairments [1]. These latter symptoms affect functioning and quality of life, contribute strongly to disability [2], and have now become primary therapeutic targets [3] because of their strong association with functional outcomes [4,5]. We have previously demonstrated both behavioral gains and improved neural system functioning after neuroscience-informed cognitive training in schizophrenia in both the chronic and early phases of psychotic disorders [6-13]. In young (aged 16-35 years) recent-onset individuals, our multisite double-blind randomized controlled trial showed that 40 hours of cognitive training delivered at home on a laptop resulted in significant improvement in global cognition, verbal memory, and problem-solving compared with a computer games control condition (CG) [8], with durable cognitive gains and improvement in positive symptoms at a 6 month follow-up [12]. Cognitive gains were significantly correlated with enhanced thalamic volume and thalamocortical connectivity as well as reduced cortical thinning in the frontal, temporal, parietal, and occipital lobes [14-16].

Despite the evidence that cognitive training can lead to benefits in cognition and functioning [17,18], patient adherence to a cognitive training regimen is highly variable. Training is highly effortful, requiring close attention to stimuli and response outcomes, processes that are impaired in psychotic disorders. It is also likely that motivational deficits have an impact on engagement with training [19-21], although these can respond to interventions that scaffold goal planning and reward processing [22].

### Objectives

Thus, we hypothesized that leveraging an intervention specifically targeted to improving motivated behavior would improve adherence and benefits from cognitive training in individuals with psychosis spectrum disorders. We examined the cognitive and clinical effects of supplementing a 16-week course of targeted cognitive and social cognitive training (TCT) with Personalized Real-Time Intervention for Motivational Enhancement (PRIME)—a smartphone-based app that includes a motivational coach and peer-to-peer interactions. To determine which changes in outcome measures would be attributable to the cognitive training, this intervention was compared with the cognitive and clinical effects of PRIME delivered with a CG.

As a mobile app, PRIME is designed to enhance motivation (but not cognition) by helping participants set and track SMART (Specific, Measurable, Achievable, Realistic, Timely) goals and by celebrating accomplishments. PRIME pairs participants with a motivational coach trained in cognitive behavioral therapy (CBT) to encourage and reinforce the goals. PRIME participants message and interact with their coach as well as a community of peers. PRIME was developed at the University of California, San Francisco, in partnership with the design company IDEO, by Danielle Schlosser [23]. It uses a user-centered design, developed with a cohort of individuals living with schizophrenia and their families who provided insight into specific values that drive one to make improvements in their quality of life. The stakeholders also provided important feedback on each feature of the app in terms of acceptability and impact on motivated behavior. A single-arm feasibility study of PRIME showed 100% retention across the intervention, with users logging in several times a week, and favorable ratings of features and overall satisfaction [23]. In a larger randomized waitlist-controlled study of young people with schizophrenia, the PRIME group showed significant improvements in social motivation, depression, defeatist beliefs, and self-efficacy relative to the waitlist group [22].

Here, we describe the procedures for this study, which was conducted entirely remotely across the United States and internationally before and during the COVID-19 pandemic; the features of the interventions; the feasibility and acceptability of the intervention in 100 randomized participants; and the results of this randomized controlled trial on cognition, motivation, symptoms, and functioning. Our primary hypotheses were as follows: (1) TCT+PRIME participants would show greater gains in cognition relative to CG+PRIME and (2) both groups would show improvements in motivation and related indices of motivation due to the effects of PRIME. Exploratory analyses tested the effects of these interventions on symptoms and functioning.

## Methods

### Study Design and Timeline

In this recently completed double-blind randomized controlled trial (ClinicalTrials.gov: NCT02782442), participants were randomized to PRIME+30 hours of TCT or PRIME+30 hours of CG in a 1:1 ratio. Before enrolling participants, a random allocation sequence was created using a randomization generator. The sequence was stored in Box and concealed or inaccessible to study members blinded to the group assignment (ie, PRIME coaches and assessment staff). The sequence was accessible to our unblinded study coordinator who enrolled and randomized the participants.

All of the study activities were completed remotely. Both groups used the PRIME app on their personal smartphones and completed cognitive training exercises or the CG on laptops or computers. The participants were asked to complete the following schedule of study activities: baseline assessments (weeks 0-3), intervention (weeks 4-20), postintervention assessments (weeks 21-23), and a 6-month follow-up assessment (weeks 49-50). The total time for study completion was approximately 1 year. Diagnosis was confirmed at baseline using the Structured Clinical Interview for Diagnostic and Statistical Manual of Mental Disorders, 5th Edition (DSM-5), Research Version (SCID-5-RV) and conducted through videoconferencing. Assessments of symptoms, functioning, motivation, and cognition at all 3 time points were also completed entirely remotely via videoconference, self-report surveys, and web-based cognitive batteries (refer to the “Measures” section for details). In total, 85% (85/100) of the participants enrolled before the COVID-19 pandemic and 15% (15/100) enrolled during the pandemic. During the COVID-19 pandemic, there was a decrease in enrollment and greater attrition during baseline assessments (ie, before randomization). Our final sample consisted of 100 randomized participants, which was a decrease from our original goal of 120 randomized participants.

### Study Population

The study sample comprised individuals aged 18 to 59 years with a psychosis spectrum disorder (refer to the inclusion criteria in the next paragraph). Participants were recruited through advertisements on Craigslist, Reddit, Schizophrenia.com [24], and the National Alliance on Mental Illness websites or were self-referred to our research group. Physical flyers and brochures were distributed in community programs and events and at the University of Minnesota clinics.

All participants met the following inclusion criteria: aged 18 to 60 years; clinical diagnosis of schizophrenia, schizoaffective disorder, schizophreniform disorder, psychotic disorder not otherwise specified, major depressive disorder with psychotic features, or bipolar disorder with psychotic features confirmed by the SCID-5-RV; fluent in spoken and written English; can demonstrate adequate decisional capacity to make a choice about participating in the research study as demonstrated by the University of California San Diego Brief Assessment of Capacity to Consent and in the judgment of the consenting study staff member; in good general physical health; outpatient status without hospitalization at least 1 month before participation; has maintained a stable dose of psychiatric medication for at least 1 month before participation; has a personal smartphone and access to a computer; willing to share contact information for their clinical provider; and if from a non–English-speaking country, the study team must be able to establish a plan for communicating with the participant’s clinical provider. Exclusion criteria were participation in research or therapy involving cognitive training within the past 3 years, a history of severe substance use in the past 3 months determined by DSM-5 criteria, and a history of neurological disorder.

### TCT and CG

#### Overview

This study used 2 computerized programs provided by Posit Science Inc through their BrainHQ portal: a TCT module focused on auditory processing and social cognition and a CG module. The CG module consists of computer games developed by Novel Games that are engaging and enjoyable but not designed to drive neuroplastic change. Participants were asked to use their assigned program (treatment or active control) on a computer for approximately 2 hours per week over the course of 16 weeks.

#### Targeted Cognitive and Social Cognitive Training

##### Auditory Training Module

This suite of exercises has been extensively studied by us and has been described in detail by Fisher et al [6]. It was designed to improve the speed and accuracy of auditory information processing while engaging working memory and cognitive control under conditions of close attention and reward. Exercises continuously adjust the difficulty level according to user performance to maintain an approximately 80% correct performance rate.

##### Social Cognition Training Module

This training module consists of exercises designed to ameliorate core deficits in social cognition expressed in schizophrenia [25]. The exercises apply principles of implicit learning to restore the brain’s capacity to process and use socially relevant information and include training to improve affect perception (both visual and vocal), social cue perception (in faces, gazes, social situations), theory of mind, self-referential style, and emotion labeling and working memory. This module has been previously studied by us, and it drives improvements in social cognition as well as measures of motivated behavior [13,25,26]. Refer to Multimedia Appendix 1 for a list of the auditory and social cognition training exercises.

#### Description of CG

CG participants rotated through a series of 13 different enjoyable commercially available computer games (eg, checkers, solitaire, crossword puzzles; Multimedia Appendix 2), playing 4 to 5 games on any given day. Games that have shown to provide face-valid cognitive stimulation and that are rated E (for everyone) by the Entertainment Software Rating Board were chosen. The CG condition was designed to control for computer exposure, contact with research personnel, and monetary payments. The CG condition is administered in exactly the same way as the TCT: the number, availability, and time spent on each game is managed by the same server that manages the treatment group exercises to match the experience between the 2 conditions.

### Description of PRIME

**Figure 1:**
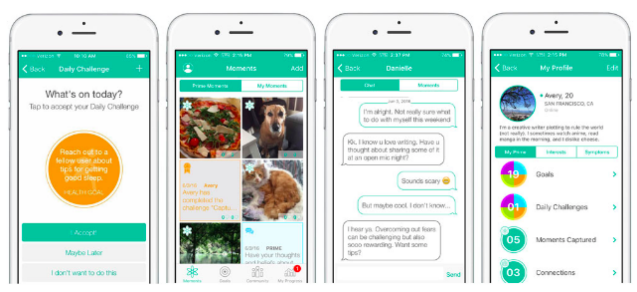
Example screenshots of the Personalized Real-Time Intervention for Motivational Enhancement (PRIME) app (from left to right): goal setting, community feed, coaching and social networking (messaging with coaches and peers), and personalized profile and metrics.

PRIME is a mobile app designed to support individuals with psychosis to manage negative symptoms and engage in motivated behavior. PRIME coaches were graduate students in counseling, clinical psychology, or social work programs, and some coaches had completed a master’s or higher degree. All coaches had previous training in CBT, motivational interviewing, or Dialectical Behavior Therapy and participated in regular study team meetings supervised by a licensed clinical psychologist (coauthor AM). PRIME techniques include CBT, behavioral activation, mindfulness, psychoeducational approaches, and coaching behaviors associated with increases in overall engagement [23]. This model incorporates a casual, collaborative tone for conversations and texts; short individual messages; and sharing of resources, including short videos and psychoeducation materials, while emphasizing behavioral strategies for any asynchronous exchanges. Text exchanges rely on communication cues that differ from face-to-face interactions [27], often creating ambiguity in meaning and tone. In light of this, PRIME uses an overtly warm tone, including expressive punctuation and positive emojis, aligning with the growing literature on messaging, behavioral change, and preferences found in other clinical populations [28,29] (Figure 1).

**Figure 2:**
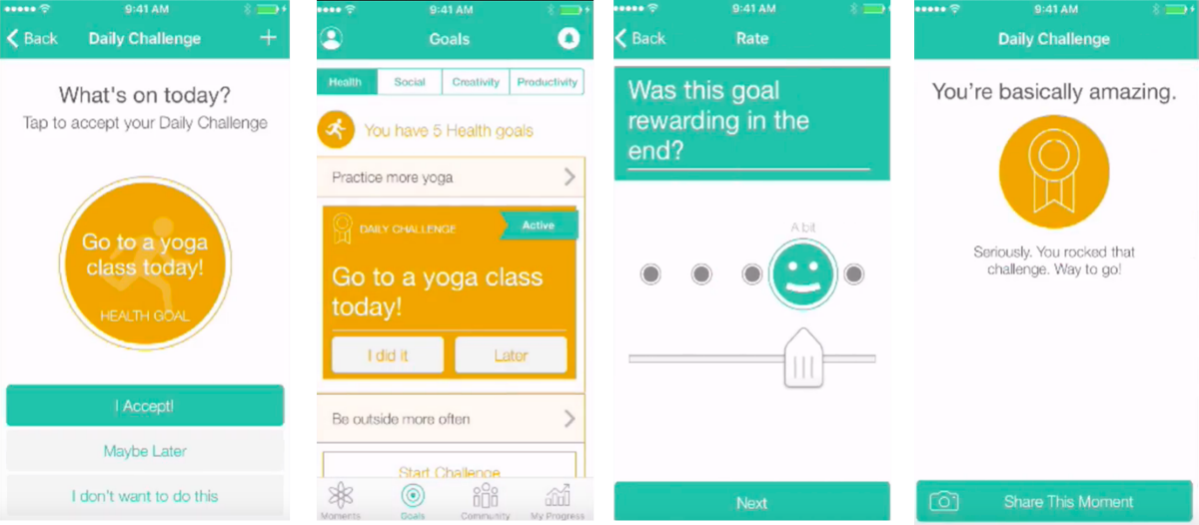
Examples of the Challenges feature (left to right): goal selection, reminders, feedback, and share with community.

Users create a profile by describing interests and symptoms and selecting long-term goals across social, body (ie, physical health), productivity, and creativity domains. While using the app, users identify which of their goals they would like to work on and are prompted with brief challenges—SMART activities related to the selected goal (Figure 2). Challenges are framed as behavioral experiments; users log predictions about the effort and pleasure associated with an upcoming challenge to compare it with what was actually exerted and derived.

**Figure 3:**
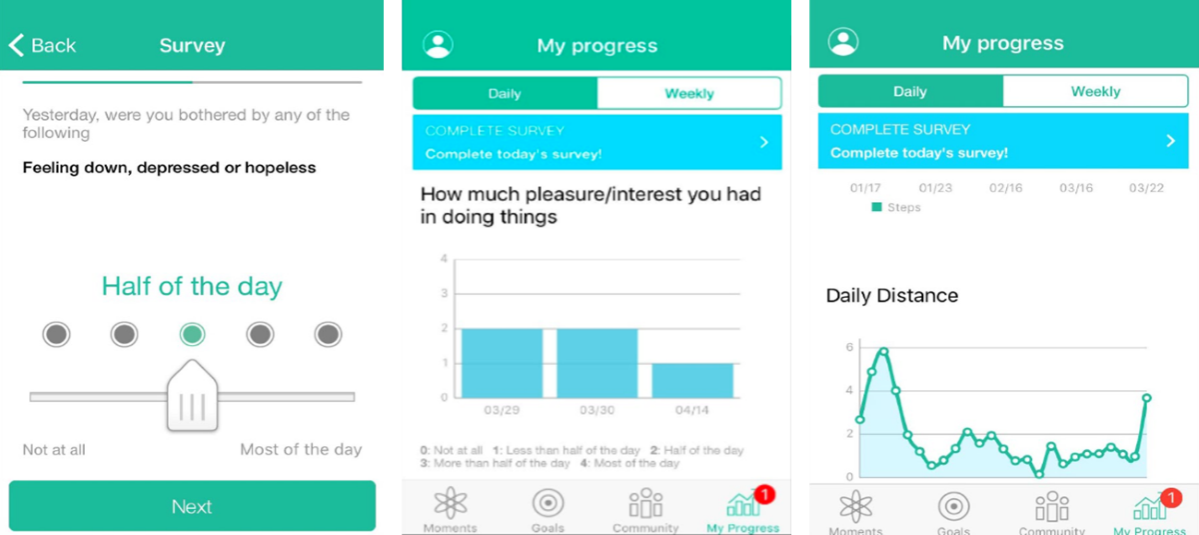
Examples of the My Progress feature (left to right): mood survey and mood and physical activity visualizations.

PRIME also helps track changes in mood, symptoms, and activity levels by pairing visualizations of surveys with motion and fitness data piped from built-in iOS and Android apps (Figure 3). Users complete the Patient Health Questionnaire-9 weekly and answer a personalized question such as “How well did I sleep last night” or “How well did your social interactions go?” each day. These visualizations can serve as feedback to help determine which challenges or other behavioral strategies are having a positive impact.

Reflecting tenets of the recovery model of psychosis [30,31], users can also interact with a community of peers with similar diagnoses, sharing direct messages as well as offering encouragement by “liking” and commenting on community posts. Each day, an automatically generated post such as “Share a silly selfie!” or “Write down a list of things you are grateful for” encourages participants to share responses. When a participant completes a challenge, an accomplishment post is automatically generated, creating additional opportunities for social reinforcement.

### Measures of Feasibility and Acceptability

The feasibility was investigated through attrition rates and use patterns on each platform. For TCT and CG, use was defined as the total hours of training completed and training intensity (hours of training completed per week). Acceptability was measured through participant ratings of satisfaction, interest, and enjoyment in PRIME and in the TCT and CG conditions and the anticipated benefits of the TCT and CG conditions. Accuracy in TCT exercises was used as an indicator of engagement and to screen for participants who were not meaningfully participating. For PRIME, use patterns include log-in frequency, number of user-initiated interactions with a motivational coach, number of peer interactions, number of community posts, number of goals set, and number of goals achieved.

### Cognition, Motivation, Symptom, and Functioning Measures

Cognition was measured using the Penn Computerized Neurocognitive Battery, Measurement and Treatment Research to Improve Cognition in Schizophrenia version. The participants were provided a link to the battery and completed the measures independently. The following self-report measures of motivation and related constructs were completed on the web via REDCap (Research Electronic Data Capture; Vanderbilt University): Motivation and Pleasure Scale (MAPS) Self Report, Motivation State Questionnaire (MSQ), Defeatist Beliefs Scale, Temporal Experience of Pleasure Scale (TEPS), Behavioral Inhibition Scale and Behavioral Activation Scale, Beck Depression Inventory, and University of California Los Angeles Loneliness Scale. Symptoms and functioning were assessed using the following interview-rated measures conducted via videoconference: Quick Scale for the Assessment of Negative Symptoms (QSANS) and Quick Scale for the Assessment of Positive Symptoms (QSAPS), the abbreviated Quality of Life Scale (QLS), and the Role Functioning Scale (RFS). Across all measures, higher scores indicated more of the construct measured. For example, higher *z* scores on cognitive measures or higher scores on motivation measures indicated better cognitive performance or greater motivation, higher ratings on symptom measures indicated greater symptom severity, and higher ratings on functioning measures indicated better functioning.

Assessors who completed the diagnostic interview and the interview-rated measures of symptoms and functioning had completed a master’s or doctoral degree in psychology or related fields and completed extensive training including observation of videos and practice ratings, observing assessments conducted by senior staff members, mock interviews, and assessment of participants while being observed by a senior staff member (coauthors MF or AC). Participants, PRIME coaches, and assessment staff were blinded to group assignment. The study coordinator was unblinded to enroll and randomize participants, perform weekly check-ins with participants, and provide support while the participants completed their assigned training program.

### Statistical Analyses

Independent samples *t* tests (2-tailed) or chi-squared tests were used to compare groups in terms of demographic variables; attrition rates; cognitive training metrics (ie, total hours of training completed and training intensity defined as hours of training per week); PRIME use (eg, number of log-ins, coach and peer interactions, and goals achieved); and participant ratings of satisfaction, interest, and enjoyment in the cognitive training and PRIME. An intent-to-treat analysis was conducted using a linear mixed-effects model, with group and time as fixed factors. The model parameters were estimated using restricted maximum likelihood. Participant groups were compared in terms of changes in the Penn cognition *z* scores and measures of motivation, symptoms, and functioning. Effect sizes (Cohen *d*) were calculated using the mean changes from baseline to posttraining and baseline to 6-month follow-up and the pooled SDs. In measures where the main effects of time or group-by-time interactions were statistically significant, post hoc contrasts were conducted from baseline to posttraining and from baseline to 6-month follow-up. All variables were screened, and outlying values less than −2.5 SD and greater than +2.5 SD from the mean were winsorized. The number of TCT participants with an accuracy threshold of ≥50% was used as an indicator of engagement and to screen for participant guessing on the cognitive training exercises. All analyses were performed using SPSS Statistics version 28 (IBM Corp).

### Ethical Considerations

The institutional review board of the University of Minnesota reviewed and approved this study (1607S90202). All participants provided informed consent. Data from this study have been deidentified. Participants in this study received US $100 for completing the diagnostic interview and baseline assessments, US $5 per session of cognitive training or computer games completed, US $55 for postintervention assessments, and US $55 for 6-month follow-up assessments.

## Results

### Participant Demographics and Locations

There were no significant differences in the demographic or clinical characteristics between the TCT+PRIME and CG+PRIME groups (Table 1). The age range of the participants was 18 to 60 years, with a slight majority of participants identifying as female (58/100, 58%). The most common diagnoses were schizoaffective disorder (42/100, 42%) and schizophrenia (38/100, 38%). The average onset of symptoms was 19.76 (SD 8.02) years, and the average number of hospitalizations was 4.30 (SD 5.53).

**Figure 4:**
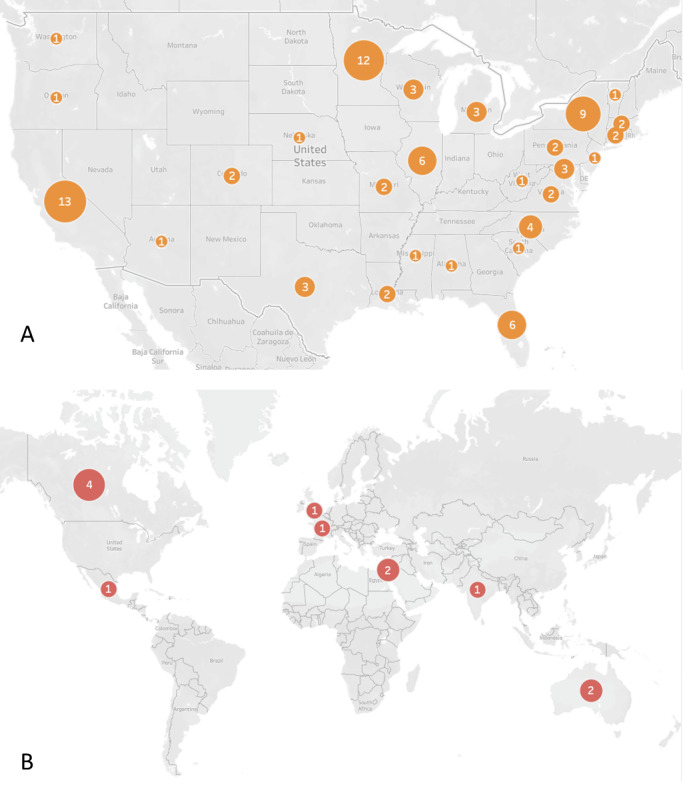
(A) Locations of participants by state and (B) locations of participants outside the United States. Numbers indicate how many participants reside in each area. Image created with Tableau [32].

Participants resided in 27 states nationally and 7 countries internationally (Figure 4).

**Table 1:** Demographic and clinical characteristics (N=100).

			TCT^a^ + PRIME^b^ (n=48)		CG^c^ + PRIME (n=52)		*t* or *χ*^2^ *(P* value*)*	
Age (years)^d^, mean (SD)			33.98 (10.60)		33.58 (10.89)		0.19 (.85)	
Female^e^, % (n/N)			56.3 (27/48)		59.6 (31/52)		0.12 (.73)	
Education (years)^d^, mean (SD)			15.47 (3.00)		15.65 (2.52)		−0.33 (.74)	
**Racial background, n (%)^e^**								3.87 (.57)
	American Indian or Alaska native	0 (0)		1 (2)				
	Asian	5 (10)		7 (13)				
	Black or African American	8 (17)		6 (12)				
	Native Hawaiian or other Pacific Islander	2 (4)		0 (0)				
	White	31 (65)		34 (65)				
	>1 race	2 (4)		3 (6)				
	Declined to answer	0 (0)		1 (2)				
**Diagnosis^e^, n (%)**								1.98 (.85)
	Schizophrenia	19 (40)		19 (37)				
	Schizoaffective	21 (44)		21 (40)				
	Schizophreniform	1 (2)		1 (2)				
	Psychosis (not otherwise specified)	1 (2)		2 (4)				
	Bipolar	3 (6)		7 (13)				
	Major depressive disorder with psychotic features	3 (6)		2 (4)				
Age of first symptoms^d^, mean (SD)			19.65 (8.77)		19.87 (7.35)		0.14 (.89)	
Total hospitalizations^d^, mean (SD)			4.23 (6.63)		4.37 (4.34)		−0.12 (.90)	

^a^TCT: targeted cognitive and social cognitive training.

^b^PRIME: Personalized Real-Time Intervention for Motivational Enhancement.

^c^CG: computer games control condition.

^d^*t* test for equality of means.

^e^Pearson chi-square.

### Participant Retention

**Figure 5:**
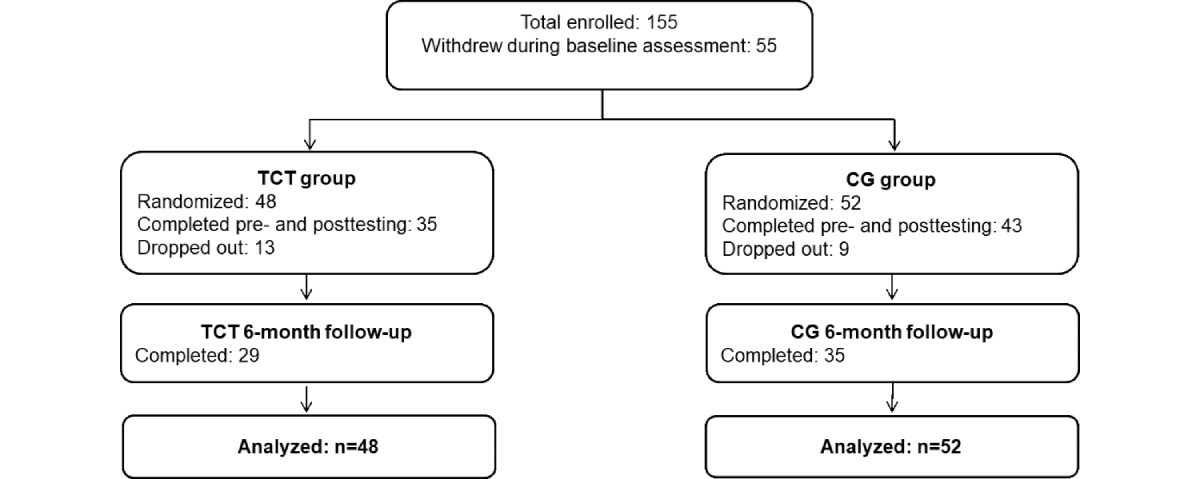
CONSORT (Consolidated Standards of Reporting Trials) diagram of study participants. CG: computer games control condition; TCT: targeted cognitive and social cognitive training.

As a measure of acceptability, retention was computed as the percentage of participants who engaged in TCT or CG plus PRIME and who completed baseline and posttraining assessments. The total attrition rate was 22% (22/100). Of the 48 participants in the TCT+PRIME condition, 35 (73%) completed the posttraining assessments, and 13 (27%) did not complete them; of the 52 participants in the in the CG+PRIME condition, 43 (83%) completed the posttraining assessments, and 9 (17%) did not complete them (Figure 5). Most participants who did not complete the posttraining assessment were lost to follow-up (ie, could not be reached after multiple attempts). The between-group difference was not significant (N=100; χ^2^_1_=1.4; *P*=.24).

### TCT and CG Use Metrics

Participants in the TCT+PRIME group averaged 17.15 (SD 13.89) hours of cognitive training across the study, with an intensity of 1.33 (SD 0.98) hours per week. Participants in the CG+PRIME group completed an average of 19.91 (SD 10.98) hours of computer games, with an intensity of 1.86 (SD 1.52) hours per week. The difference between the groups was not statistically significant for total hours of training (t_98_=1.10; *P*=.28) but was significant for intensity (t_98_=2.05; *P*=.04). The average accuracy in TCT exercises was 69.6% (SD 7.04%), with only 1 participant scoring <50% accuracy.

### PRIME Use Metrics

Across both conditions, participants logged into PRIME an average of 63.98 (SD 34.10) times, on average 4 to 5 times per week. The log-in rate significantly decreased from the first half of participation (up to week 8) to the second half (t_96_=4.56; *P*<.001), although the average change was less than 1 fewer log-ins per week (mean change 0.83, SD 1.79). In total, participants shared 7612 interactions (messages, comments, and “likes”) with their coach and 887 interactions between peers. CG+PRIME participants initiated more coach interactions relative to TCT+PRIME, and this difference was at a trend level (*P*=.06), although it was not statistically significant. The average number of community posts per participant was 21.84 (SD 25.59). The average number of goals set per participant was 14.98 (SD 17.03), and the average number of goals achieved was 14.16 (SD 14.66) or 95% (14.16/14.98). The most popular goals were related to physical health and well-being (body), with participants setting an average of 6.93 (SD 7.51) body goals. The number of goals set related to social activities, productivity, and creativity averaged 2 to 3 per category. There were no statistically significant differences in any PRIME use metrics between the TCT+PRIME and CG+PRIME groups (Table 2).

**Table 2:** Personalized Real-Time Intervention for Motivational Enhancement (PRIME) use metrics (N=96)^a^.

Outcome measures			TCT^b^ + PRIME (n=45), mean (SD)		CG^c^ + PRIME (n=51), mean (SD)		*t* (*P* value)
Number of log-ins			64.44 (30.66)		63.57 (37.18)		0.13 (.90)
Total weeks of use			13.78 (4.80)		13.67 (6.00)		0.10 (.92)
Proportion of log-ins			0.67 (0.22)		0.67 (0.24)		0.10 (.92)
Log-ins per week			4.69 (1.54)		4.73 (1.62)		−0.13 (.90)
Log-ins per week (first half)			5.27 (1.64)		5.06 (1.97)		0.59 (.56)
Log-ins per week (second half)			4.35 (2.24)		4.31 (2.24)		0.09 (.93)
Number of interactions with coach			61.13 (59.39)		95.22 (107.03)		−1.89 (.06)
Number of interactions with peers			8.47 (13.48)		9.49 (18.75)		−0.30 (.76)
Community posts			20.44 (24.44)		23.08 (26.75)		−0.50 (.62)
Spontaneous posts			3.24 (6.54)		5.53 (11.29)		−1.19 (.24)
Achievement posts			16.02 (19.67)		16.78 (18.44)		−0.20 (.85)
**Goals set**			13.78 (16.95)		16.39 (17.15)		−0.86 (.39)
	Social	2.02 (3.26)		2.75 (3.58)		−1.03 (.31)	
	Body	6.84 (8.10)		7.00 (7.02)		−0.10 (.92)	
	Productivity	2.09 (3.86)		2.94 (4.08)		−1.05 (.30)	
	Creativity	2.42 (4.15)		3.71 (5.18)		−1.33 (.19)	
Goals achieved			11.87 (12.36)		16.18 (16.27)		−1.45 (.15)

^a^Due to technical difficulties, PRIME use metrics were unavailable for 4 participants.

^b^TCT: targeted cognitive and social cognitive training.

^c^CG: computer games control condition.

### The Effects on Cognition

Statistically significant main effects of time were observed in global cognition, attention, and emotion recognition, with both groups showing improvements (refer to Table 3 and Multimedia Appendix 3 for *P* and Cohen *d* values from baseline to posttraining and baseline to follow-up).

There was a statistically significant group-by-time interaction in emotion recognition and a non–statistically significant trend in global cognition, with the TCT+PRIME group showing greater improvements relative to the CG+PRIME group (Table 3). In emotion recognition, within-group comparisons showed significant gains in the TCT+PRIME group from baseline to posttraining (*P*=.05; Cohen *d*=0.37) and from baseline to follow-up (*P*=.007; Cohen *d*=0.56) and significant gains in the CG+PRIME group from baseline to posttraining (*P*=.001; Cohen *d*=0.55) but no significant change from baseline to follow-up (*P*=.28; Cohen *d*=0.19). In global cognition, within-group contrasts showed significant gains in the TCT+PRIME group from baseline to posttraining (*P*=.002; Cohen *d*=0.61) and nonsignificant improvement from baseline to follow-up (*P*=.15; Cohen *d*=0.28), and nonsignificant change in the CG+PRIME group from baseline to posttraining (*P*=.54; Cohen *d*=0.10) and from baseline to follow-up (*P*=.66; Cohen *d*=0.08).

**Table 3:** *z* scores on cognitive and social cognitive outcome measures at baseline, posttraining, and at 6-month follow-up in the targeted cognitive and social cognitive training (TCT) + Personalized Real-Time Intervention for Motivational Enhancement (PRIME) and computer games control condition (CG) + PRIME groups.

Outcome measures^a^	TCT + PRIME (n=48)				CG + PRIME (n=52)				Main effects of time, *P* value		Group × time interaction, *P* value
	Baseline, mean (SE)	Posttraining, mean (SE)	6-month follow-up, mean (SE)	Baseline mean (SE)		Posttraining, mean (SE)	6 month follow-up, mean (SE)				
Global cognition	0.04 (0.09)	0.33 (0.10)	0.24 (0.12)	−0.03 (0.09)		0.004 (0.09)	0.01 (0.11)	.03		.09	
Motor speed	−0.02 (0.17)	0.28 (0.20)	0.34 (0.18)	0.27 (0.16)		0.17 (0.18)	0.37 (0.16)	.17		.38	
Speed of processing	0.41 (0.09)	0.50 (0.09)	0.47 (0.08)	0.34 (0.08)		0.34 (0.08)	0.47 (0.07)	.33		.53	
Attention	−0.07 (0.16)	0.24 (0.14)	0.35 (0.08)	−0.20 (0.15)		0.04 (0.12)	0.27 (0.07)	<.001		.72	
Working memory	−0.40 (0.18)	0.04 (0.25)	−0.33 (0.30)	−0.25 (0.17)		−0.50 (0.22)	−0.34 (0.27)	.83		.29	
Verbal learning	−0.22 (0.18)	−0.04 (0.21)	−0.46 (0.30)	−0.26 (0.17)		−0.45 (0.19)	−0.63 (0.27)	.14		.44	
Verbal memory	−0.40 (0.17)	−0.21 (0.23)	−0.50 (0.28)	−0.41 (0.16)		−0.59 (0.20)	−0.50 (0.26)	.84		.40	
Visual learning	−0.32 (0.17)	−0.01 (0.20)	−0.27 (0.22)	−0.31 (0.16)		−0.30 (0.18)	−0.39 (0.20)	.37		.68	
Visual memory	−0.37 (0.16)	−0.32 (0.21)	−0.10 (0.21)	−0.44 (0.16)		−0.29 (0.19)	−0.33 (0.20)	.33		.66	
Problem-solving	0.29 (0.16)	0.53 (0.18)	0.36 (0.19)	0.39 (0.15)		0.35 (0.16)	0.08 (0.17)	.10		.37	
Emotion recognition	0.79 (0.19)	1.13 (0.16)	1.19 (0.20)	−0.11 (0.18)		0.39 (0.14)	0.14 (0.18)	.005		<.001	

^a^Penn Computerized Neurocognitive Battery: global cognition (composite *z* score across all measures), motor speed (Motor Praxis Test), speed of processing (Digit Symbol Test), attention (Short Continuous Performance Test), working memory (Short Letter N-Back Test), verbal learning (Word Memory Test), verbal memory (Word Memory Delayed Test), visual learning (Short Visual Object Learning Test), visual memory (Short Visual Object Learning Delayed Test), problem-solving (Matrix Analysis Test), and Emotion Recognition test.

### The Effects on Motivation Indices

In the total sample, statistically significant main effects of time indicated improvements in MAPS social pleasure, MAPS work and recreation, MAPS motivation to engage in activities, MSQ overall motivation, MSQ self-efficacy, defeatist beliefs, and TEPS consummatory pleasure (Table 4; Multimedia Appendix 3).

There was a statistically significant group-by-time interaction in MSQ Extrinsic Motivation. Within-group comparisons showed nonsignificant change in the TCT+PRIME group from baseline to posttraining (*P*=.48; Cohen *d*=0.13) and from baseline to follow-up (*P*=.87; Cohen *d*=0.03). The CG+PRIME group showed nonsignificant change from baseline to posttraining (*P*=.18; Cohen *d*=0.23) and a significant decrease from baseline to follow-up (*P*=.04; Cohen *d*=0.37). All other group-by-time interactions were not significant.

**Table 4:** Scores on motivation indices, symptom ratings, and functional outcomes at baseline, posttraining, and at 6-month follow-up in the targeted cognitive and social cognitive training (TCT) + Personalized Real-Time Intervention for Motivational Enhancement (PRIME) and computer games control condition (CG) + PRIME groups.

Outcome measures			TCT + PRIME (n=48)							CG + PRIME (n=52)							Main effects of time, *P* value			Group × time interaction, *P* value
			Baseline, mean (SE)		Posttraining, mean (SE)		6-month follow-up, mean (SE)		Baseline, mean (SE)			Posttraining, mean (SE)		6-month follow-up, mean (SE)						
**Motivation indices**																				
	MAPS^a^ social pleasure	1.94 (0.15)		2.25 (0.17)		2.18 (0.18)		1.99 (0.15)			2.26 (0.16)		2.01 (0.16)		.02			.82		
	MAPS work and recreation	2.17 (0.16)		2.58 (0.17)		2.42 (0.16)		2.03 (0.15)			2.35 (0.15)		2.28 (0.14)		.02			.79		
	MAPS motivation about close relationships	2.12 (0.15)		2.34 (0.17)		2.26 (0.20)		2.11 (0.14)			2.22 (0.15)		2.17 (0.17)		.33			.94		
	MAPS motivation to engage in activities	1.90 (0.14)		2.15 (0.16)		2.07 (0.16)		1.78 (0.13)			2.09 (0.14)		2.07 (0.14)		.04			.91		
	MSQ^b^ overall motivation	2.85 (0.15)		3.14 (0.16)		3.29 (0.18)		3.08 (0.14)			3.24 (0.14)		3.23 (0.16)		.04			.53		
	MSQ intrinsic motivation	2.98 (0.14)		3.18 (0.16)		3.07 (0.18)		3.15 (0.14)			3.20 (0.15)		3.25 (0.16)		.47			.74		
	MSQ extrinsic motivation-pressure	1.97 (0.15)		2.13 (0.18)		2.01 (0.17)		2.51 (0.14)			2.27 (0.16)		1.98 (0.15)		.13			.04		
	MSQ self-efficacy	2.83 (0.16)		2.87 (0.19)		3.13 (0.20)		2.86 (0.15)			2.98 (0.17)		3.17 (0.18)		.03			.98		
	Defeatist beliefs	3.28 (0.17)		3.09 (0.18)		3.18 (0.18)		3.34 (0.16)			3.10 (0.17)		3.03 (0.17)		.05			.74		
	TEPS^c^ anticipatory pleasures	4.15 (0.15)		4.18 (0.18)		4.25 (0.18)		4.34 (0.15)			4.38 (0.17)		4.31 (0.17)		.92			.61		
	TEPS consummatory pleasure	4.25 (0.15)		4.45 (0.18)		4.34 (0.20)		4.37 (0.14)			4.65 (0.16)		4.36 (0.18)		.02			.64		
	Behavioral Inhibition Scale	1.87 (0.08)		1.86 (0.10)		1.99 (0.11)		1.72 (0.08)			1.85 (0.09)		1.78 (0.10)		.32			.19		
	BAS^d^ reward responsivity	1.60 (0.07)		1.56 (0.09)		1.66 (0.11)		1.67 (0.07)			1.65 (0.08)		1.69 (0.10)		.56			.85		
	BAS drive	2.16 (0.10)		2.17 (0.12)		2.12 (0.14)		2.23 (0.09)			2.03 (0.11)		2.16 (0.12)		.37			.33		
	BAS fun seeking	2.05 (0.09)		2.13 (0.12)		2.15 (0.13)		2.02 (0.08)			1.98 (0.11)		1.92 (0.11)		.94			.52		
**Symptom ratings**																				
	QSANS^e^	32.49 (2.87)		26.31 (3.17)		25.75 (3.20)		31.98 (2.76)			30.39 (2.95)		31.75 (2.97)		.09			.31		
	QSAPS^f^	16.58 (1.86)		17.92 (2.24)		16.62 (2.02)		18.05 (1.79)			18.67 (2.08)		14.36 (1.85)		.04			.48		
	Beck Depression Inventory	20.68 (2.19)		19.47 (2.56)		18.61 (2.42)		24.02 (2.08)			18.01 (2.29)		18.63 (2.15)		.05			.42		
	UCLA^g^ Loneliness Scale	2.28 (0.11)		2.29 (0.12)		2.36 (0.14)		2.26 (0.10)			2.40 (0.11)		2.30 (0.12)		.46			.46		
**Functional outcomes**																				
	QLS^h^ social functioning	2.91 (0.26)		3.13 (0.30)		3.24 (0.27)		2.84 (0.25)			3.12 (0.28)		3.06 (0.25)		.14			.95		
	QLS occupational functioning	3.77 (0.31)		3.43 (0.33)		3.45 (0.36)		3.25 (0.30)			3.57 (0.31)		3.54 (0.34)		.99			.13		
	QLS intrapsychic foundations	3.90 (0.14)		4.16 (0.16)		4.12 (0.16)		3.94 (0.13)			3.99 (0.15)		3.83 (0.15)		.26			.43		
	QLS environmental engagement	5.58 (0.08)		5.75 (0.10)		5.75 (0.11)		5.58 (0.08)			5.42 (0.09)		5.45 (0.10)		.97			.04		
	QLS mean item total	3.85 (0.14)		4.04 (0.16)		4.05 (0.15)		3.80 (0.13)			3.90 (0.15)		3.81 (0.14)		.22			.59		
	RFS^i^ work productivity	4.67 (0.24)		4.39 (0.25)		4.61 (0.25)		4.62 (0.23)			4.79 (0.24)		4.68 (0.23)		.89			.25		
	RFS independent living	5.25 (0.19)		5.23 (0.20)		5.28 (0.20)		5.25 (0.18)			5.28 (0.19)		5.38 (0.19)		.8			.98		
	RFS family network	5.73 (0.17)		5.53 (0.20)		5.58 (0.21)		5.96 (0.16)			5.72 (0.18)		5.54 (0.20)		.04			.47		
	RFS social network	4.56 (0.26)		5.02 (0.28)		4.87 (0.27)		4.92 (0.25)			4.87 (0.26)		4.93 (0.25)		.46			.53		

^a^MAPS: Motivation and Pleasure Scale.

^b^MSQ: Motivation State Questionnaire.

^c^TEPS: Temporal Experience of Pleasure Scale.

^d^BAS: Behavioral Activation Scale.

^e^QSANS: Quick Scale for the Assessment of Negative Symptoms.

^f^QSAPS: Quick Scale for the Assessment of Positive Symptoms.

^g^UCLA: University of California Los Angeles.

^h^QLS: Quality of Life Scale.

^i^RFS: Role Functioning Scale.

### The Effects on Symptoms and Functioning

In the total sample, statistically significant main effects of time indicated improvements in the Beck Depression Inventory, QSAPS, and improvement at the trend level (although this was not statistically significant) in the QSANS (Table 4; Multimedia Appendix 3). In the RFS Family Network, a significant main effect of time indicated a decline in the total sample.

There was a significant group-by-time interaction in the QLS Environmental Engagement subscale. Within-group comparisons showed significant improvement in the TCT+PRIME group from baseline to posttraining (*P*=.02; Cohen *d*=0.43) and from baseline to follow-up (*P*=.03; Cohen *d*=0.42). The CG+PRIME group showed a significant decrease from baseline to posttraining (*P*=.05; Cohen *d*=0.31) and a decrease at the trend level, although this was not statistically significant, from baseline to follow-up (*P*=.09; Cohen *d*=0.30). All other group-by-time interactions were not significant.

### Satisfaction, Interest, and Enjoyment With TCT, CG, and PRIME

In total, 72% (72/100) of the participants evaluated the TCT and CG modules and the features of PRIME during an exit interview (Table 5). When asked about attitudes and experiences regarding cognitive training or computer games on a Likert scale ranging from 0 (none of the time) to 4 (all the time), participants rated interest in cognitive training and computer games similarly (difference in means=0.02; *P*=.94). Participants in the TCT group rated the exercises as more difficult than those in the CG group (difference in means=0.72; *P*=.004); however, the TCT group tended to find the instructions for cognitive training easier to understand compared with the CG group (difference in means=0.39; *P*=.07). In terms of anticipated impact of the exercises (“The training will help me do well at school or work”), participants in the TCT group rated cognitive training as slightly more meaningful relative to the CG group, although this difference was not statistically significant (difference in means=0.46; *P*=.15).

Regarding overall satisfaction with PRIME, the TCT and CG groups responded similarly, with a mean rating of 7.74 (SD 2.05) on a scale of 1 (completely disagree) to 10 (completely agree). Among features, participants rated satisfaction with daily discussion topics and the relatability of coach community posts slightly more highly compared with helpfulness of the mood tracking feature. There were no significant differences between the groups.

**Table 5:** Participants’ ratings of targeted cognitive and social cognitive training (TCT), computer games control condition (CG), and Personalized Real-Time Intervention for Motivational Enhancement (PRIME).

Outcome measures			TCT + PRIME (n=33), mean (SD)		CG + PRIME (n=39), mean (SD)		*t* (*P* value)
**The training^a^**							
	...is interesting	2.03 (1.05)		2.05 (1.34)		0.07 (.94)	
	...is enjoyable	1.88 (1.05)		2.21 (1.36)		1.15 (.26)	
	...is easy	1.97 (1.08)		2.69 (1.00)		2.95 (.004)	
	...is effective	2.06 (1.27)		1.97 (1.29)		−0.28 (.78)	
	...is having a positive effect on daily life	2.22 (1.50)		1.82 (1.32)		−1.19 (.24)	
	...will improve my thinking and memory	2.38 (1.29)		2.05 (1.34)		−1.03 (.31)	
	...will help me do well at school or work	2.25 (1.24)		1.79 (1.38)		−1.44 (.15)	
	The training sessions felt helpful to me	2.12 (1.34)		1.87 (1.32)		−0.79 (.43)	
	The training instructions are easy to understand	3.52 (0.76)		3.13 (0.98)		−1.85 (.07)	
	I felt I was making progress	2.30 (1.05)		2.08 (1.28)		−0.80 (.43)	
**PRIME^b^**							
	I felt satisfied with the daily discussion topics	7.00 (2.70)		7.16 (2.50)		0.26 (.80)	
	Moments posted by the coaches felt relatable to me	7.30 (2.54)		7.44 (2.23)		0.24 (.81)	
	Tracking my mood in PRIME was helpful	7.00 (2.91)		6.82 (3.16)		−0.25 (.81)	
	Overall satisfaction with PRIME	7.73 (2.04)		7.74 (2.09)		0.03 (.97)	

^a^Each statement was rated on a scale ranging from 0 to 4 (0=none of the time, 1=a little bit of the time, 2=about half the time, 3=most of the time, and 4=all of the time).

^b^Each statement was rated on a scale ranging from 1 (completely disagree) to 10 (completely agree).

## Discussion

This is the first randomized controlled trial to explore how cognitive training, supported by a novel smartphone-based app designed to improve motivational deficits, can be delivered remotely to individuals with psychosis spectrum disorders. All procedures were conducted entirely online, enabling participation from individuals both nationally and internationally.

### Participants

The participants in this study were on average aged 34 (SD 10.70) years, 35% (35/100) were people of color, 58% (58/100) identified as women, and 46% (46/100) had an average of several years of postsecondary education (mean of 15.57 years of education in the total sample, SD 2.75). This is in contrast to our prior studies of TCT in adults with persistent schizophrenia spectrum disorders where participants were recruited from community outpatient mental health clinics, where the average age was 43 (SD 12.85) years, 50% were people of color, 28% identified as women, and the average number of years of education was 13.5 (SD 2.24) [13,33]. These results indicate that web-based recruitment results in a sample that is younger, less racially diverse, with a larger number of women, with greater number of years of education, and with average cognitive performance relative to studies using traditional brick-and-mortar recruitment methods from community clinics.

This is consistent with the study by Moseson et al [34] who compared 3 studies on cognitive health, diabetes, and hypertension that used web-based recruitment methods with 19 studies in the same fields that used traditional recruitment methods (eg, in-person screening and consent in clinics). The web-based recruitment studies had slightly younger participants, more female participants, and were split on enrollment of racial minorities, with some of the web-based recruitment studies showing less diversity relative to the traditional recruitment studies and others showing more diversity. Web-based recruitment also resulted in a more geographically diverse sample and faster recruitment. Education level was not compared in this study.

These and our results suggest that although there are some benefits to web-based recruitment (eg, an increase in recruitment of women, a more geographically diverse sample, and increased efficiency), there are also limitations. Web-based recruitment led to a less racially diverse sample in our study and in some of the studies by Moseson et al [34]. Moseson et al [34] suggested that investigators should use this knowledge to carefully consider strategies for increasing outreach and recruitment of individuals from underrepresented populations. Participants in our study also showed greater years of education and were required to have a personal smartphone and access to a computer. Although we did not compare socioeconomic status, it is likely that web-based recruitment may have led to a sample with higher socioeconomic status that had greater access to digital devices. Future studies should consider loaning devices to increase the recruitment of diverse individuals.

### Intervention Adherence

The dropout rate of this study was 22% (22/100), which is lower than that in our previous studies of TCT in adults with persistent schizophrenia and with recent-onset psychotic disorders where training was completed remotely (where it was 30% [12]). Previous studies on TCT social cognition exercises in adults with psychosis spectrum disorders that also included group therapy and peer-to-peer messaging showed similarly low dropout rates (22% [35] and 20% [26]), whereas studies involving PRIME alone have shown dropout rates <10% [22,23]. These results suggest that the use of socially supportive and interactive therapeutic interventions such as PRIME may increase adherence to cognitively demanding and effortful treatments such as TCT.

Although retention in both the TCT and CG groups was higher in this study than in similar studies without PRIME, the TCT participants showed a lower weekly training intensity, likely reflecting the greater effort and difficulty of engaging in TCT exercises. The completion of the training remotely may have also contributed to the lower training intensity. Our prior study of TCT completed in person in the research setting showed an average training intensity of approximately 5 hours per week [33], whereas prior studies of TCT cognitive or social cognitive exercises completed remotely show training intensities ranging from <1 hour per week [26] to approximately 3 hours per week [12,13.

To retain blinding, the coaches were instructed to redirect discussions that might reveal specific details of the specific computer module a participant was engaged in (TCT or CG). Although critical to the design of a randomized controlled trial, this practice created obstacles to meaningful conversations about the effortful nature of cognitive training and inhibited motivational troubleshooting regarding the training itself. In a separate study of first-episode psychosis, we are currently investigating the effects of cognitive training provided with PRIME versus treatment as usual using a regression discontinuity design. In this study, discussion of the training between participants, coaches, and peers is encouraged and embedded in the treatment planning for the individual [36].

### PRIME Use

Most participants used PRIME multiple times per week with only a slight drop as the study progressed, a finding that was consistent between the TCT and CG groups. Although direct messaging with a coach was by far the most used feature of PRIME, users frequently expressed value and inspiration in peer interactions and peer community posts. Body and creativity goals (averaging 6.86 and 3.07 goals set, respectively) were the most popular categories selected by participants, whereas social goals (averaging 2.40 goals set) were the least popular category. This is noteworthy considering how impairments in social functioning are often referenced as primary treatment goals of those living with psychosis spectrum disorders [37], where defeatist beliefs and negative appraisals often exacerbate actual performance difficulties and social aversion [38-40]. Future research can examine ways to harness popular PRIME body goals such as “go on a walk” to create low-risk social interactions.

### TCT+PRIME Show Durable Gains in Emotion Recognition

Our results indicate significantly greater improvement in emotion recognition, and greater improvement at the trend level, although this was not statistically significant, in global cognition in the TCT+PRIME group relative to the CG+PRIME group. In emotion recognition, both groups showed improvement from baseline to posttraining; however, these effects were only durable at the follow-up in the TCT+PRIME group, with a medium effect size. In global cognition, the total sample showed a significant gain; however, within-group contrasts revealed that only the TCT+PRIME group showed improvement from baseline to posttraining, with an effect size in the medium range, whereas the CG+PRIME group showed negligible change. At follow-up, the gains in the TCT+PRIME group showed some durability with an effect size in the small range; however, these results did not reach statistical significance. Both groups also showed durable gains in attention, likely owing to the attentional demands of the TCT and CG exercises.

The TCT+PRIME group results are consistent with those of our prior study of cognitive training combined with social cognition training versus cognitive training alone (TCT only) in 111 participants with schizophrenia spectrum disorders [13]. Both groups showed improvements in cognition and symptoms; however, the cognitive training combined with social cognition training group showed greater improvement in social cognition and reward processing relative to the TCT-only group. Effect sizes on social cognitive measures were larger in this prior study and likely owing to the greater number of hours of training completed (70 hours recommended vs 30 hours recommended in this study). Although the effects were not as large in this study, our findings suggest that durable gains in emotion recognition can be achieved with substantially fewer hours of training.

Our prior studies on cognitive training versus a CG in chronic schizophrenia and recent-onset schizophrenia have also shown larger effects at posttraining and follow-up [7,12]. Again, this could be because of the greater number of hours of training completed in these studies (ie, 40-50 recommended hours). However, this is also likely owing to differences in our samples. The sample in this study did not show cognitive deficits at baseline and had an average of 15 years of education, whereas in our prior studies, participants showed baseline global cognition approximately 1 SD below the mean of healthy controls and an average of 13 years of education. This is consistent with a meta-analysis [17] that found that individuals with schizophrenia with lower premorbid IQ, fewer years of education, and greater baseline symptom severity showed greater gains from cognitive training, likely because there is more room for improvement.

Finally, the differences in these effects across studies could also be the result of differences in the measures. In our prior studies, the MATRICS Consensus Cognitive Battery was administered in person [12,13,33]. In this study, a computerized battery of measures analogous to the MATRICS Consensus Cognitive Battery was used and completed remotely.

### PRIME Provides Durable Improvements in Motivation and Related Constructs

The total sample showed improvements in a majority of the motivational domains, and these improvements were sustained at the 6-month follow-up. These included changes in experiential pleasure (MAPS social pleasure, MAPS work and recreation, and TEPS consummatory pleasure), MAPS motivation to engage in activities, and ratings of overall motivation; improvement in feelings of self-efficacy; and a reduction in defeatist beliefs. Notably, these benefits also appear to be reflected in the negative symptom measures (QSANS, Beck Depression Inventory; refer to the “PRIME Induces Improvement in Symptoms” section). These results are consistent with a prior study of PRIME alone (ie, no cognitive training) in 38 young people with schizophrenia [22]. Participants in the PRIME condition showed significantly greater improvements in self-efficacy, defeatist beliefs, and depression, and improvements at the trend level, although these were not statistically significant, on the MAPS relative to a waitlist control condition.

There was a significant group-by-time interaction in the MSQ Extrinsic Motivation subscale, with nonsignificant change in the TCT+PRIME group and a significant decrease in the CG+PRIME group from baseline to follow-up. This subscale measures feelings of pressure from friends or family to make improvements in one’s social life, academic or work life, and general health. The CG+PRIME group ratings indicated feeling less external pressure at the 6-month follow-up, whereas the TCT+PRIME group showed no significant change. It is possible that feelings of external pressure may not have changed in the TCT+PRIME group because of the demands of the cognitive training program. In our exit interview, the TCT+PRIME group rated the exercises as significantly more difficult than the CG+PRIME group. This is consistent with a recent study of BrainHQ exercises versus computer control games in survivors of breast cancer [41]. Cognitive training was seen as challenging, engaging, and gave a sense of accomplishment, whereas control games were seen as a way of taking mind off issues, enjoyable, and easy to navigate. The barriers to cognitive training included an awareness of failing, whereas control games were deemed to be too repetitive.

### PRIME Induces Improvement in Symptoms

Symptoms improved over time in the total sample. Specifically, symptom reductions were observed in the Beck Depression Inventory and QSAPS, whereas the QSANS showed a decrease at the trend level, although this was not statistically significant, indicating improvement in depressive, positive, and negative symptoms. This pattern is consistent with our previous studies of computerized cognitive training in individuals with chronic schizophrenia [13] and recent-onset schizophrenia [12] where reductions in symptoms were evident at a 6-month follow-up in those who completed the cognitive training. What is unique about this study compared with previous studies is that the control group did not receive cognitive training, but it still showed a modest improvement in symptoms. This difference may be attributable to the PRIME app, as both groups had access to the resources on the app. Our results on depressive symptom reduction have been found in previous research comparing PRIME with treatment as usual or waitlist [22]. Finally, meta-analyses of cognitive training in psychosis have shown comparable small effect sizes at posttraining on symptoms [17,42] but not at follow-up [42].

The overall pattern of most of the functional outcome measures showed no evidence of a meaningful difference, which parallels the findings of our previous studies [7,12,13]. Two exceptions were found in the QLS Environmental Engagement subscale and the RFS Family Network. The QLS Environmental Engagement subscale showed a significant group-by-time interaction, with the TCT+PRIME group showing improvement and the CG+PRIME group showing a decline from baseline to posttraining and from baseline to follow-up. However, there were no statistically significant changes in the main functional outcomes of the QLS (ie, social and occupational functioning). The RFS Family Network showed a decline across the total sample. Most of the decrease occurred from baseline to posttraining while participants were using the PRIME app. Increased engagement with the PRIME community may have led to a decline in family involvement. Furthermore, although the change was significant, the mean rating at follow-up was still in the moderate range, indicating that participants, on average, were still seeing a family member more than once a month across all time points.

Improving everyday functioning likely requires both cognitive training and functional skills training [43]. Future studies are needed to determine the effects of remotely delivered cognitive training provided with remotely delivered psychosocial treatments. A recent review and meta-analysis of remotely delivered, evidence-based psychosocial treatments for schizophrenia spectrum disorders found a limited number of studies, with effect sizes on functioning in the small range, and similar to the effect sizes of these treatments when provided in person [44]. One promising approach is metacognitive training, which combines psychoeducation, cognitive bias modification, and strategy teaching, with effect sizes ranging from small to moderate on functioning, and evidence of durable improvements in symptoms and functioning up to 1 year postintervention [45].

### Satisfaction, Interest, and Enjoyment With Cognitive Training and PRIME

Responses between the TCT and CG groups regarding specific training experiences were not significantly different, with the exception of ease of training, which was rated more highly by the CG group. Although participants were typically neutral in their reports of enjoyment and interest in the exercises, cognitive training appeared to be engaging enough to avoid random guessing, with only 1 participant falling below the 50% accuracy. Group differences were observed for questions probing immediate or anticipated impact of the training (eg, “The training will help me do well at school or work.”) that tended to elicit higher ratings in the TCT group, although these differences were not statistically significant. Participants in both groups tended to rate the features of PRIME favorably, averaging 7.7 out of 10 in overall satisfaction.

Previous studies of cognitive training in other diagnoses have also found that participants’ ratings of control and active cognitive training conditions are similar in terms of enjoyment and engagement and in terms of perceived cognitive gains [46,47]. Keefe et al [46] noted that increasing the enjoyability of digital training interventions could lead to greater adherence and greater cognitive gains. In turn, daily functioning could be affected by encouraging efforts to deploy those improved cognitive skills. In a recent commentary, Harvey [48] notes this as potentially very important, given that improvement in objective cognitive performance and subjective assessments of performance are associated with improvements in everyday functioning [49]. Thus, important future directions include (1) increasing the enjoyability of digital cognitive training interventions and (2) providing interventions, such as metacognitive training, that might increase participants’ insights into cognitive improvements and encouragement of the use of these improvements in daily functioning.

### Strengths and Limitations

The strengths of this study include our relatively large sample size and racial, geographic, clinical, and gender diversity. PRIME is an innovative digital intervention targeting domains in psychotic disorders that have few alternative treatments currently available. All elements of the study were fully remote and delivered to English-speaking individuals in 8 different countries. The browser interface of cognitive training and the mobile interface of PRIME make both interventions highly scalable, although access to a laptop, a smartphone, and the internet are required. The remote nature of the interventions also created limitations in assessing the quality of engagement with TCT or CG, as we were unable to assess active and distracted engagement or random inputs (although this was likely not the case, as only 1 participant showed a cognitive training accuracy level <50%). Additional limitations arise from our sample, which was recruited on the web and consisted of highly educated participants with average baseline cognitive performance. Future studies should consider strategies to increase the recruitment of a more diverse sample.

### Conclusions

This study aimed to identify and inform scalable remote interventions that address both cognitive and motivational impairments in psychosis using neuroscience-informed cognitive training exercises and a novel smartphone-based app. Our retention rate of 78% (78/100) and the national and international geography of participation in our study indicate that this approach is acceptable, feasible, tolerable, and scalable and that cognitive training and the PRIME app can induce improvements in emotion recognition, motivation, and symptoms that persist 6 months after the interventions. Future analyses will test models of the relationship between hours of cognitive training completed; PRIME use; and changes in cognition, motivation, symptoms, and functioning.

## Appendix

Multimedia Appendix 1 Cognitive and social cognitive training exercises.

Multimedia Appendix 2 Commercially available computer games.

Multimedia Appendix 3 Effect sizes (Cohen d) from baseline to posttraining and baseline to 6-month follow-up in measures showing statistically significant or trend-level, but not significant, main effects of time.

Multimedia Appendix 4 CONSORT-eHEALTH (V 1.6.1).

## Abbreviations

CBT: cognitive behavioral therapy
CG: computer games control condition
DSM-5: Diagnostic and Statistical Manual of Mental Disorders, 5th Edition
MAPS: Motivation and Pleasure Scale
MSQ: Motivation State Questionnaire
PRIME: Personalized Real-Time Intervention for Motivational Enhancement
QLS: Quality of Life Scale
QSANS: Quick Scale for the Assessment of Negative Symptoms
QSAPS: Quick Scale for the Assessment of Positive Symptoms
REDCap: Research Electronic Data Capture
RFS: Role Functioning Scale
SCID-5-RV: Structured Clinical Interview for Diagnostic and Statistical Manual of Mental Disorders, 5th Edition, Research Version
SMART: Specific, Measurable, Achievable, Realistic, Timely
TCT: targeted cognitive and social cognitive training
TEPS: Temporal Experience of Pleasure Scale

## Data Availability

The data sets generated during this study are available from the corresponding author upon reasonable request.
