# Supplementary material for: The Effects of Remote Cognitive Training Combined With a Mobile App Intervention on Psychosis: Double-Blind Randomized Controlled Trial
Source: J Med Internet Res. 2023 Nov 13;25:e48634. doi: 10.2196/48634 (PMC10682932; doi:10.2196/48634)
Supplement: Multimedia Appendix 1 [file jmir_v25i1e48634_app1.docx]

**Supplemental Table 1.** Cognitive and social cognitive training exercises.

| Exercise | Description |
| --- | --- |
| Sound Sweeps | Indicate the direction of two consecutive sound sweeps. Each one can sweep either up or down in pitch. |
| Syllable Stacks | Memorize the syllables heard and click on them in the order they were presented. The sequence becomes longer as the task progresses. |
| Fine Tuning | Select the syllable heard from a given pair. Syllables in the pair become increasingly similar as the difficulty increases. |
| Memory Grid | Match pairs of sound cards in a memory game. |
| Recognition | A face is quickly shown, followed by a series of faces. Identify the face shown. |
| Gaze Match | Match the gaze direction of a target face. |
| Face to Face | An emotion is shown, followed by a series of faces. Identify the face with the same emotion. |
| Face Facts | Memorize and recall names, faces, and facts about people. |
| Prosody | Select the emotional prosody of sentences with neutral content. |
| Prosody Theory of Mind | Select the correct vocal response given the situation described in the short audio script. |
