# Supplementary material for: The Effects of Remote Cognitive Training Combined With a Mobile App Intervention on Psychosis: Double-Blind Randomized Controlled Trial
Source: J Med Internet Res. 2023 Nov 13;25:e48634. doi: 10.2196/48634 (PMC10682932; doi:10.2196/48634)
Supplement: Multimedia Appendix 2 [file jmir_v25i1e48634_app2.docx]

**Supplemental Table 2.** Commercially available computer games.

| Exercise | Description |
| --- | --- |
| Chinese Checkers | The participant must move all their pieces to their opponent’s end by moving their pieces to any adjacent spot or “jumping” over one or more pieces already on the board. |
| Sudoku | Classic Sudoku. The object of the puzzle is to fill each square with a number from 1 to 9 such that no number is repeated in the same row, column, or 3x3 subsection. |
| Reversi | Players take turns placing disks on the board with their assigned color facing up. During a play, any disks of the opponent's color that are in a straight line and bounded by the disk just placed and another disk of the current player's color are turned over to the current player's color. The object of the game is to have the majority of disks in the participant’s color. |
| Double Klondike Solitaire | This is Classic solitaire that flips three cards at a time from the draw pile. Participants must stack cards alternating in color in descending order with the goal of forming complete A-K stacks of the same suit. |
| Tri Peaks Solitaire | The object of the game is to remove all of the cards that make up the “three peaks.” At the bottom of the screen, there is a deck with cards turned down and one card that is shown. Participants must stack the cards present on the ‘peaks’ to the card on the bottom. For example, if the card shown is the number two – the participant would be able to stack a ‘3’ or a ‘Ace,’ therefore removing those cards from the base of the peaks. |
| Brick Breaking Hex | Click on a group of blocks with the same color. At the beginning of each game, the participant has a set number of stars. To remove individual blocks, you lose one of your stars. The goal is to get rid of all the blocks before the stars are lost. |
| Brick Squasher II | Use the mouse to control the board to bounce the balls and destroy the bricks. Some bricks require a few hits and some bricks are indestructible. |
| Gem Swap | Swap adjacent gems to create 3 or more in a row to remove the gems. |
| War Ship | Participant hides ships, and then takes turns with a computer player to search for the opponent’s hidden ships. The object of the game is to find the opponent’s ships and sink them before they find and sink yours. |
| A Maze Race | There are two balls, the green one is designated to the participant and the red one is the computer player. The participant must find the ‘Flag’ or end point before the computer does. |
| Lineup 4 | Participant and computer player take turns dropping colored discs from the top into a grid. The player must connect four yellow discs in a row (vertically, horizontally or diagonally) before the opponent. |
| Word Search II | Letters are placed in a grid and the participant must find the specified list of words hidden within the grid. |
| Crossword Puzzle | The participant must hover over the line of blocks to view the hint. |
