# Supplementary material for: The Effects of Remote Cognitive Training Combined With a Mobile App Intervention on Psychosis: Double-Blind Randomized Controlled Trial
Source: J Med Internet Res. 2023 Nov 13;25:e48634. doi: 10.2196/48634 (PMC10682932; doi:10.2196/48634)
Supplement: Multimedia Appendix 3 [file jmir_v25i1e48634_app3.docx]

**Supplemental Table 3.** Effect Sizes (Cohen’s d) from baseline to post-training and baseline to 6 month follow-up in measures showing statistically significant or trend level significant main effects of time.

| **Outcome measures^a^** | **Baseline to Post-Training** | | **Baseline to 6 Month Follow-Up** | |
| --- | --- | --- | --- | --- |
|  | Main Effect of Time *P* value | Effect Size (Cohen’s d) | Main Effect of Time *P* value | Effect Size (Cohen’s d) |
| **Cognition** | | | | |
| **Global Cognition** | .008 | 0.30 | .11 | 0.17 |
| **Attention/Vigilance** | .03 | 0.25 | <.001 | 0.46 |
| **Emotion Recognition** | .001 | 0.48 | .03 | 0.30 |
| **Motivation Indices** | | | | |
| **MAPS Social Pleasure** | .005 | 0.33 | .28 | 0.07 |
| **MAPS Work and Recreation** | .005 | 0.30 | .03 | 0.21 |
| **MAPS Motivation to Engage in Activities** | .01 | 0.24 | .04 | 0.23 |
| **MSQ Overall Motivation** | .05 | 0.26 | .01 | 0.31 |
| **MSQ Self Efficacy** | .56 | 0.11 | .01 | 0.34 |
| **Defeatist Beliefs** | .04 | 0.22 | .04 | 0.21 |
| **TEPS Consummatory Pleasure** | .03 | 0.27 | .70 | 0.06 |
| **Symptom and Functional Outcomes** | | | | |
| **QSANS** | .05 | 0.26 | .06 | 0.23 |
| **QSAPS** | .46 | 0.08 | .21 | 0.10 |
| **Beck Depression Inventory** | .02 | 0.23 | .02 | 0.23 |
| **RFS Family Network** | .08 | 0.23 | .01 | 0.32 |
